# Differential energetic profile of signal processing in central vestibular neurons

**DOI:** 10.3389/fneur.2024.1469926

**Published:** 2024-11-25

**Authors:** Lars Kunz, Hans Straka

**Affiliations:** Division of Neurobiology, Faculty of Biology, Ludwig-Maximilians University Munich, Planegg, Germany

**Keywords:** ATP, tonic, phasic, firing pattern, amphibian, metabolism, movement, potassium channels

## Abstract

**Background:**

Energetic aspects of neuronal activity have become a major focus of interest given the fact that the brain among all organs dominates the oxygen consumption. At variance with the importance of neuroenergetics, the knowledge about how electrical activity and metabolism is correlated in defined neuronal populations is still rather scarce.

**Results:**

We have estimated the ATP consumption in the two physiologically well characterized populations of frog central vestibular neurons, with tonic and phasic firing patterns, respectively. These two distinct groups of neurons jointly process head/body movements detected by semicircular canal and otolith organs in the inner ear. The ATP consumption for maintenance of the resting membrane potential (V_r_) and postsynaptic action potential (AP) generation was calculated based on the wealth of previously reported morpho-physiological features of these two neuronal types. Accordingly, tonic vestibular neurons require less ATP across the physiological activity range for these major processes, than phasic vestibular neurons, despite the considerably higher firing rates of the former subtype. However, since both neuronal subtypes are indispensable for the encoding and processing of the entire head/body motion dynamics, the higher energy demand of phasic neurons represents an obvious and necessary price to pay. Although phasic and tonic neurons form the respective core elements of the frequency-tuned vestibular pathways, both cellular components are cross-linked through feedforward and feedback side loops. The prominent influence of inhibitory tonic neurons in shaping the highly transient firing pattern of phasic neurons is cost-intensive and contributes to energy consumption for electrical activity in addition to the already extensive energy costs of signal processing by the very leaky phasic vestibular neurons.

**Conclusion:**

Despite the sparse production of action potentials by phasic vestibular neurons, the computation by this neuronal type dominates the ATP expense for processing head/body movements, which might have contributed to the late evolutionary arrival of this central neuronal element, dedicated to the encoding of highly dynamic motion profiles.

## Introduction

Detection and adequate processing of head motion signals are a prerequisite for a constant maintenance of visual acuity and postural stability as well as for updating subcortical and cortical circuits dedicated to navigation and orientation in space (1). Head motion signals are detected by semicircular canal and otolith organs in the inner ear, transduced into electrical activity by hair cells and relayed as sequences of action potentials by VIIIth nerve afferent fibers that terminate on neurons in the hindbrain vestibular nuclei (2–4). These central vestibular neurons constitute the key elements for all vestibulo-motor transformations (5) and for the distribution of body motion-related signals to other centers in, e.g., the cerebellum and cortex (6).

Naturally occurring head/body motions exhibit a large dynamic bandwidth. For head angular velocity, values up to 1,000 deg./s in *Xenopus laevis* tadpoles (7), 1,300 deg./s in mice (8), 1,500 deg./s in macaque monkey (8) and 410 deg./s in humans (9) were reported. Linear head acceleration can reach values up to 4.5 G in mice (8), 8 G in macaque monkeys (8) and 4.1 G in humans (9). This precludes signal processing by a simple set of prototypic central vestibular neurons (10, 11). Rather, all vertebrates investigated so far possess two dynamically different vestibular neuronal types (12). The nomenclature of these subtypes, though, differs between species. In rodents and guinea pigs, they are called type A and B neurons [e.g., (13, 14)], in the cat kinetic and tonic neurons (15), in chicken principal and elongate cells (16), and in frogs phasic and tonic neurons (17). Nonetheless, the common denominator that derives from all studies is an apparent duality of central vestibular neurons with respect to intrinsic membrane properties and emerging signal processing capabilities (see (12)).

**Figure 1:**
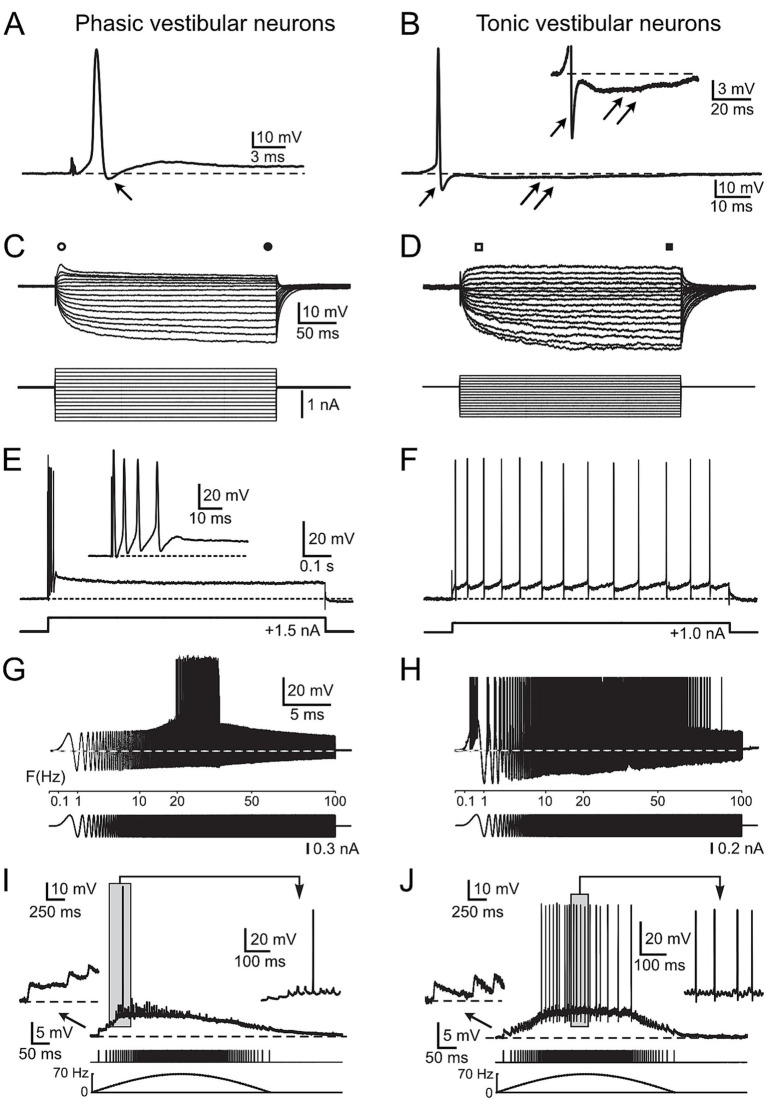
Physiological characteristics of frog phasic and tonic central vestibular neurons. (A, B) Action potentials of phasic (A) and tonic (B) second-order vestibular neurons (2°VN); differences include the presence of a monophasic after-hyperpolarization (AHP) in phasic (single arrow in A) and a biphasic AHP in tonic 2°VN (single and double arrows in B); inset in B illustrates the AHP at higher magnification. (C,D) Voltage responses (upper traces) to series of hyper- and depolarizing current steps (lower traces) in a phasic (C) and a tonic (D) 2°VN; phasic but not tonic 2°VN exhibit an initial transient in response to positive current steps (open circle and square) that decreases to lower values during the subsequent plateau phase (filled circle and square). (E,F) Spike burst discharge in phasic 2°VN (E) and continuous spike firing in tonic 2°VN (F) during intracellularly injected positive current steps. (G,H) Spike discharge (upper traces) evoked by injection of sinusoidally modulated currents that increase in frequency up to 100 Hz (lower traces) in a phasic (G) and a tonic 2°VN (H); note the band-pass and low-pass firing rate range of phasic and tonic 2°VN, respectively. (I,J) Subthreshold compound responses and spikes in a phasic (I) and a tonic (J) 2°VN after stimulation with sinusoidally modulated pulse trains at a peak frequency of 70 Hz; the first three EPSPs and the spikes during the indicated period (gray area) are shown at higher magnification, respectively; bottom traces indicate the pulse train and stimulus frequency (dashed lines). A–F with permission from Straka et al. (17); G, H with permission from Beraneck et al. (34); I, J with permission from Pfanzelt et al. (21).

Based on the experimental advantages of isolated whole-brain preparations with functionally intact central circuits and vestibular afferent inputs through individual branches of the VIIIth nerve [e.g., (18)], electrophysiological studies in frogs have been particularly successful in delineating those intrinsic membrane and synaptic properties that assign to tonic and phasic vestibular neurons low- and band-pass filter properties, respectively (19). The clear duality of vestibular neurons, reported for adult frogs (Figure 1), and the wealth of available knowledge on morpho-physiological details [e.g., (14, 19–21)] make them particularly suitable for the attempt to calculate the energetic running costs of the two neuron types. Utilizing these parameters in a mathematical model (22–24) allows to link intrinsic membrane and synaptic response properties of the two sets of frog central vestibular neurons with the estimated energetic costs of the respective neuronal computations. Similar leaky neurons (i.e., with a relatively low membrane resistance in the range of 10–20 MOhms) in the mammalian auditory brainstem (22, 25, 26) exhibited high energetic costs for their electrical activity. Therefore, our expectation was that the running costs of phasic central vestibular neurons in frogs might also be rather high. In a multi-step approach, we applied the model to passive and active intrinsic membrane properties, spike generation, processing of synaptic sensory information as well as multisynaptic integration of excitatory and inhibitory signals. Thereby, we were able to calculate the respective ATP consumption of the different physiological elements to run the complexity of neuronal computations in the two vestibular neuron types. The known spike discharge pattern evoked by naturalistic afferent activity patterns along with the delineated synaptic circuitry facilitated a direct reference between the obtained energetic cost and the neuronal transformation of vestibular signals.

## Methods

### Calculation of neuronal ATP consumption

The employed mathematical model calculates the ATP consumption of the Na^+^/K^+^ ATPase to restore the Na^+^ gradient across the plasma membrane, which is perturbed either by action potentials (APs) or by constantly active ion channels responsible for the input resistance (R_in_) and homeostasis of the resting membrane potential (V_r_) (22–24). The calculation of the ATP consumption for V_r_ maintenance (E_Vr_) only requires V_r_ and R_in_ to be experimentally obtained (Equation 1; Table 1).


(1) $$E_{Vr}=\frac{I_{Na}}{3\;F}=N_{L}\cdot \frac{\left(E_{Na}-V_{r}\right)\cdot \left(V_{r}-E_{K}\right)}{\left(F\cdot R_{\mathrm{in}}\left(V_{r}+2E_{Na}+3E_{K}\right)\right)}$$

with F = Faraday constant = 96,485C mol^−1^, I_Na_ = Na^+^ flux, N_L_ = Avogadro constant = 6.022e23 mol^−1^, E_Na_ = +59 mV, E_K_ = −98 mV, V_r_ = resting membrane potential, R_in_ = input resistance.

**Table 1:** Electrophysiological and morphological parameters used in Equations 1, 2.

	V_r_ (mV)	R_in_ (MOhm)	O (μm^2^)	ΔAP (mV)
Phasic	−69,7	13,9	1,479	58,9
Tonic	−68,6	25,1	1,006	56,5
Reference	(17)	(17)	(24)	(17)

The reversal potentials of Na^+^ and K^+^, also included in the calculation, were set to E_Na_ = 59 mV and E_K_ = −98 mV based on values reported for frog neurons (27). The number ‘3’ in the equation is based on the three Na^+^ pumped in one cycle that consumes one ATP molecule.

For the calculation of the ATP cost for AP generation (E_AP_), only two more neuronal properties, the cell surface area, O, and the voltage change during the AP relative to V_r_ (ΔAP), have to be considered (Equation 2; Table 1).


(2) $$E_{AP}=f\cdot EF\cdot \frac{n\left(Na\right)}{3}=f\cdot EF\cdot \frac{Q}{\left(3e\right)}=f\cdot EF\cdot C\cdot \frac{\Delta AP}{\left(3e\right)}=f\cdot EF\cdot O\cdot C_{S}\cdot \frac{\Delta AP}{\left(3e\right)}$$

with C = membrane capacitance, C_s_ = specific membrane capacitance = 1e-14F · μm^−2^, ΔAP = AP amplitude relative to V_r_, EF = efficiency factor = 2 (22, 24), f = firing frequency, n(Na) = amount of Na^+^ (in mol), O = cell surface, Q = membrane charge, e = elementary charge = 1.6e-19C.

**Figure 2:**
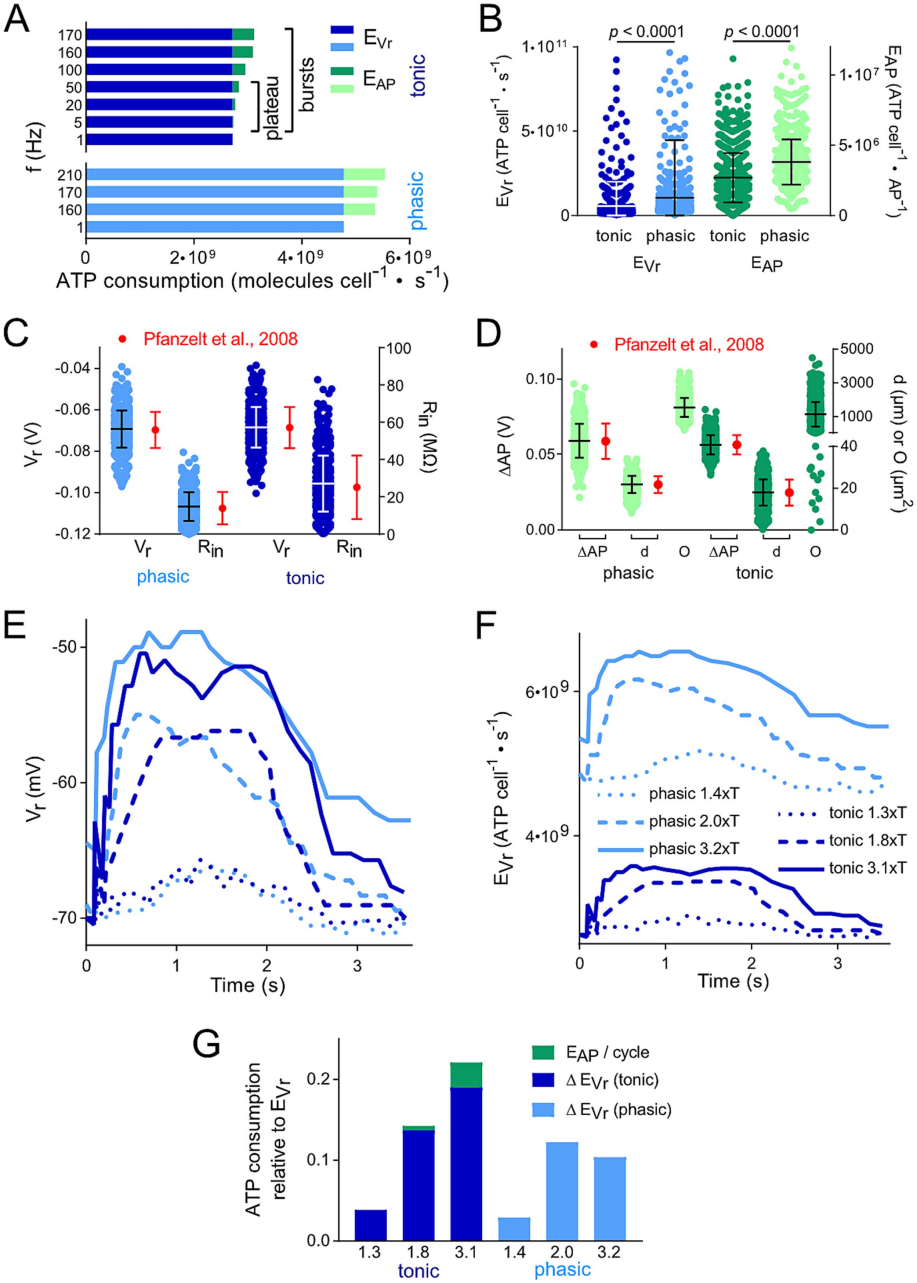
Membrane polarization level and thus spike firing frequency affects calculated ATP consumption. (A) Comparison of the energetic cost for action potential generation (E_AP_) and resting membrane potential maintenance (E_Vr_) of tonic and phasic vestibular neurons for different frequencies of APs; for tonic neurons, both the firing rates during the initial burst and the plateau phase were considered. (B) Distribution of E_Vr_ and E_AP_ for 1,000 individual cells calculated from random data for resting membrane potential (V_r_), input resistance (R_in_), voltage change during AP relative to V_r_ (ΔAP) and surface area (O) based on means ± SD of available values (17, 20). (C,D) Physiological distribution of parameters relevant for the calculation of the energetic cost for E_AP_ and E_Vr_. Values for V_r_, R_in_, ΔAP and d were randomly created for 1,000 cells based upon measured means ± SD and a Gaussian distribution (red dots and error bars; data from Straka et al. and (17) Beraneck et al. (20). These values represent the reference for the distribution of E_Vr_ and E_AP_ depicted in (B). Statistical significance of the differences between the two types of vestibular neurons was tested by an unpaired t-test with Bonferroni correction. (E,F) Change of V_r_ (E) and calculated E_Vr_ (F) of example tonic and phasic vestibular neurons during one pulse train cycle with a peak stimulus rate of 70 Hz and varying stimulus strengths (in multiples of the threshold T; for labeling of traces see F). Time course of V_r_ was obtained from Figures 5A–F in Pfanzelt et al. (21). Calculated E_Vr_ in F represent the basis for the data in G. (G) Comparison of the total ATP consumption (E_Vr_ and E_AP_) for the neurons analyzed in (C,D); ATP consumption is shown relative to the ATP consumption for maintaining V_r_ during a period without electrical stimulation of afferent inputs. Numbers on the x axes represent the same multiples of T as in E and F.

As in most studies, we approximated the area O as the somatic surface of a sphere calculated from the mean soma diameter available from morphological studies (20). In recent studies, the efficiency factor EF was set to 1–2 dependent on the cell type (24). We chose a value of 2 like in our study on auditory brainstem neurons (22), because they share the low input resistance R_in_ as major trait with the vestibular neurons. Changing EF from 2 to 1 would halve the values of E_AP_ (Equation 2). However, this would have little influence on the total ATP consumption, since E_Vr_ was much larger than E_AP_ (Figure 2A). We saw no reason to choose different EFs for phasic and tonic vestibular neurons, because the electrophysiological parameters are rather similar (Table 1).

**Figure 3:**
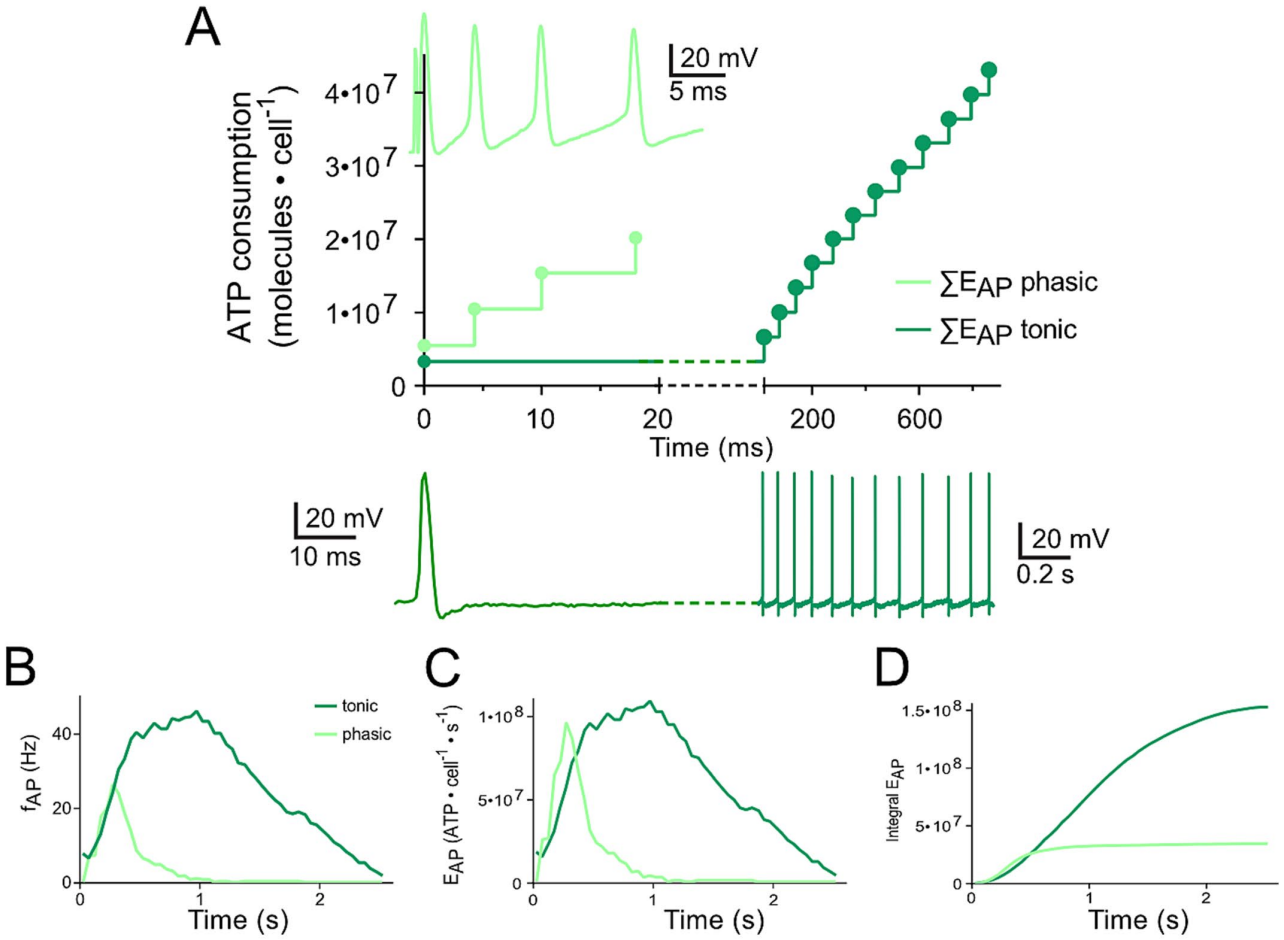
Differential changes of E_Vr_ in phasic and tonic neurons dependent on the respective firing pattern. (A) Calculated cumulative ATP consumption in temporal relation to action potential occurrence (APs; as insets) generated by phasic (light green) and tonic (dark green) vestibular neurons. The traces of the phasic (light green) and the tonic neuron (dark green), respectively, were obtained from Figures 1A6, B7 (17) and are also depicted in Figures 1E,F. (B–D) Mean firing rate over time for the two types of vestibular neurons [B; obtained from Figures 5G1,G2 (21)] as reference for calculating the time course of E_AP_ (C) and cumulative E_AP_ (D).

For calculation of the time course of ATP consumption, we have digitized the published subpanels stated in the Figure legends by means of DigitizeIt^1^ to obtain the time course of V_r_ (e.g., Figure 2E) or firing frequency (Figure 3B). Using these values, we calculated the time course of E_Vr_ (Equation 1; Figures 2F, 3A,C,D) and of E_AP_ (Equation 2; Figure 3C), respectively. We performed all calculations either in Excel (Microsoft) or in Prism (GraphPad). The statistical analysis was carried out in Prism. We used Prism and Affinity Designer (Serif) to produce the graphs of the manuscript.

### Recreation of physiological populations of vestibular neurons

With the aim to calculate ATP consumption for individual vestibular neurons, 1,000 hypothetical, but physiologically sound neurons were recreated. Therefore, we first randomly generated the parameters V_r_, R_in_, ΔAP and d (and thereby O) based on published mean values and errors and the literature-based assumption of Gaussian distributions (Figures 2C,D; Table 1) (17, 24). In a second step, E_Vr_ and E_AP_ were calculated for these individual neurons using Equations 1, 2 (Figure 2B).

## Results

### Physiological features of central vestibular neurons

Central vestibular neurons in adult ranid frogs have been characterized extensively in isolated whole brain preparations with respect to intrinsic membrane and synaptic properties (11, 17). Based on the analysis of a large number of intracellular recordings, frog vestibular neurons subdivide into two distinct populations of neurons, which differ from each other by several physiological parameters (17). Phasic vestibular neurons exhibit broader action potentials (APs) and monophasic after-hyperpolarizations (AHP, arrow; Figure 1A), lower input resistances and rectifying I-V curves (i.e., larger current responses to negative than to positive voltages; Figure 1C), initial spike bursts and subsequent lack of persistent firing upon permanent membrane depolarization (Figure 1E). These features create band-pass filter-like properties (with AP firing in the frequency band 20–30 Hz in the example; Figure 1G). They are responsible for the highly transient synaptic responses evoked by electrical pulse train stimulation of vestibular nerve afferent fibers (i.e., they fire only briefly in the beginning of the cycle with rising stimulation frequency; Figure 1I). In contrast, tonic vestibular neurons exhibit narrower APs and biphasic AHPs (Figure 1B; inset shows biphasic AHP), higher input resistances, linear I-V curves (Figure 1D) and a constant spike firing upon membrane depolarization (Figure 1F). As a consequence, they show low-pass filter-like properties (with AP firing up to 80 Hz in the example; Figure 1H). Stimulation of vestibular nerve afferent fibers result in a rather linear synaptic response profile, i.e., the AP firing frequency directly reflects the frequency of the electrical input (Figure 1J). These distinct physiological differences assign to the two vestibular subtypes different computational capabilities that derive from co-adapted cellular and circuit properties (19). Given the clear correlation between physiological features and energetic cost of the respective computations (22, 23, 28), the collectivity of available cellular and circuit characteristics of frog central vestibular neurons was fed into a mathematical model to calculate the metabolic costs of the neuronal processing by the two cell types. The description of the respective metabolic demands will be progressively increased in complexity by first focusing on the maintenance of the membrane potential and intrinsic membrane properties. This was followed by calculating the ATP demand for the differential action potential dynamics and intrinsic firing patterns, culminating in the inclusion of synaptic properties and response profiles both from vestibular nerve afferents as well as from inhibition by local and cerebellar circuits.

### Impact of intrinsic membrane properties on ATP consumption

The consumption of ATP as most important energy equivalent for fueling various aspects of the neuronal activity was calculated based on available biophysical and morphological parameters of the two neuronal subtypes (17). The aim was to compare their respective demands and to identify potential energy saving or expending mechanisms. Based on measurements and model predictions, the two major processes of ATP consumption in neurons are maintaining the resting membrane potential (E_Vr_) and generating APs (E_AP_) (23, 24). Accordingly, the underlying mathematical model (22–24, 28) was applied using Equations 1, 2 and the parameters given in Table 1 to estimate the energetic cost for these key features for each of the two vestibular cell types (Figure 2).

These calculations demonstrated that the energetic cost for maintaining the resting membrane potential (E_Vr_; 3–5 · 10^9^ molecules · cells^−1^ · s^−1^) was considerably higher when compared to the generation of APs (E_AP_; 0.5–1 · 10^9^ molecules · cells^−1^ · s^−1^) (Figure 2A). We found only minor differences in the ratio of E_Vr_ to E_AP_ for the two processes in tonic and phasic vestibular neurons. However, the excessive E_Vr_ applied to both subtypes and, thus, total energy expenditure was independent of spike firing frequencies and profiles across the respective physiological range (Figure 2A). In addition, this energetic cost distribution for neuron-specific operations in central vestibular neurons is very similar to that of cells located in adjacent hindbrain nuclei such as auditory neurons (22) and therefore potentially represents a typical feature of brainstem neurons. However, when “recreating” individual vestibular neurons with randomly distributed biophysical and morphological parameters across the physiological range (see Methods and Figures 2C,D), a surprising variability of the ATP consumption emerged during the calculation (Figure 2B). In fact, for both fractions of ATP consumption (E_AP_ and E_Vr_), the statistical test clearly revealed that the two parameters in the generated model population were substantially more energy-demanding in phasic compared to tonic vestibular neurons. This confirms the initial calculation presented in Figure 2A, simply based on mean values for intrinsic parameters and despite the same overall resting membrane potential of the two cell types (17). The higher energetic cost for maintaining the membrane potential of the leakier phasic vestibular neurons, indicated by the significantly lower input resistance (17), matches the similarly high energy demand of very leaky auditory brainstem neurons (22), but is in stark contrast to most neuronal types in the cerebral and cerebellar cortex (23, 24) (see discussion).

### Impact of synaptic activity on ATP consumption

The calculation of the energy demand for the maintenance of the resting membrane potential and production of APs in the two vestibular neuronal subtypes provided already a clear distinction with respect to computational costs simply based on intrinsic properties. However, a more naturalistic condition is represented by taking into account the physiologically occurring spike pattern of, e.g., phasic neurons, which fire only very few action potentials upon intracellular constant current injection (Figure 1E) or upon synaptic excitation following electrical activation of vestibular nerve afferents with a pulse train of varying frequency (Figure 1). We analyzed a single cycle of activity, i.e., the cyclic change in V_r_ (Figures 1I,J, 2E). As a result, the more pronounced ATP consumption of phasic neurons at each time-point was obvious (Figure 2F). In fact, E_Vr_ values, summarized over one depolarization episode, considerably exceeded the cumulated E_Vr_ values for a corresponding period without membrane polarizati on. Moreover, both, E_Vr_ and E_AP_ of tonic neurons exhibited a strong and linear relation with polarization strength (indicated as multiples of the stimulus threshold T) (Figure 2G).

The comparison of ATP consumption for AP generation is of particular interest due to the distinctly different firing patterns of the two neuronal subtypes and the differential insertion into local inhibitory circuits (21). Phasic vestibular neurons receive, apart from the monosynaptic afferent excitation, pronounced longer-latency inhibitory inputs through local interneurons (29) and cerebellar Purkinje cells (21), while tonic vestibular neurons lack such inputs altogether. The interneuron activity, which by shunting the input resistance limits AP generation in phasic neurons upon excitation (30) is likely to have a significant metabolic consequence that has to be added to the principal energy expedition of this neuronal subtype (see below). With respect to the timing, the two types of vestibular neurons produce APs with different temporal regimes during the induced membrane depolarization (Figures 3A,B), which was initially used as classifying hallmark feature. As a consequence, ATP consumption follows a very different time course in tonic and phasic vestibular neurons. Based on the spike timing, phasic neurons consume almost all ATP for AP generation during the first third of a prolonged stimulus (Figure 3C). In contrast, cumulative ATP consumption of tonic neurons being active over essentially the entire cycle of a stimulus pulse train profoundly exceeds that of phasic neurons in the time domain (Figure 3D). Thus, despite E_AP_ being smaller than E_Vr_ (Figures 2A,B), the phasic nature of the response dynamics in combination with the impact of inhibitory interneurons (see below) on the truncation of the discharge of phasic neurons during a longer-lasting activation may surprisingly represent an energy-saving mechanism in the latter subtype.

### Impact of inhibitory inputs on ATP consumption in phasic neurons

**Figure 4:**
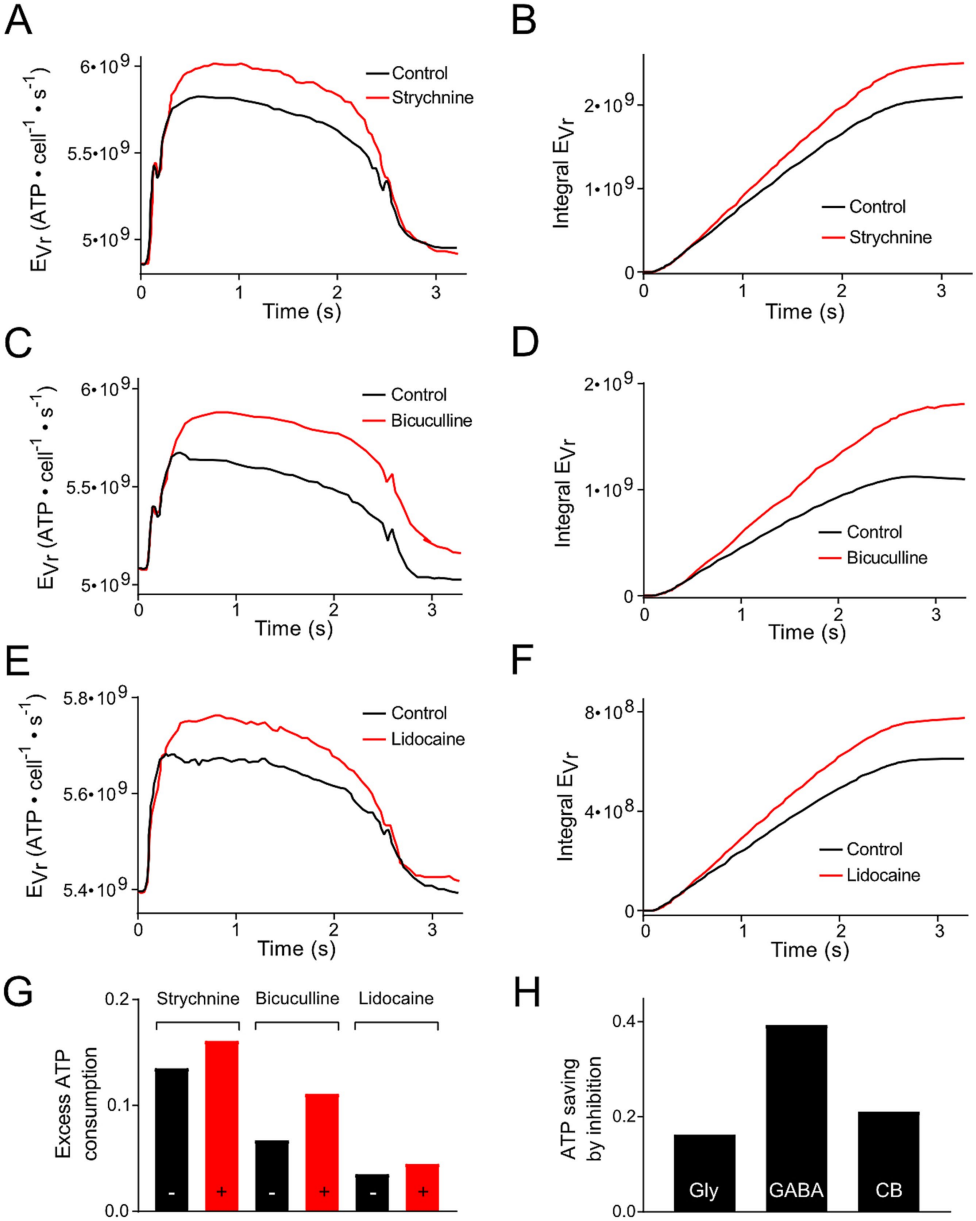
Modification of ATP consumption of phasic vestibular neurons by including inhibition mediated by local interneurons and the cerebellum. (A,C,E) Energetic cost for the resting membrane potential maintenance (E_Vr_) of phasic vestibular neurons calculated for the control condition (black traces) and following blockage of the glycinergic inhibition by strychnine (A, red trace), of the GABAergic inhibition by bicuculline (C, red trace) and of the cerebellar Purkinje cell-mediated inhibition by injection of lidocaine into the cerebellum (E, red trace); original data obtained from Figures 6A,C,E (21). (B,D,F) Cumulative ATP consumption derived from the data in (A,C,E). (G) Excess ATP consumption across a single stimulus cycle for the data presented in (A–F) calculated as fraction of the ATP consumption across a period without stimulation. (H) Calculated ATP saving due to the inhibition based on the data depicted in (G).

Inhibitory inputs, relayed onto phasic vestibular neurons might represent a highly relevant factor for the energy cost of this subtype for three major reasons:

(1) Pfanzelt et al. concluded that inhibitory currents attenuate changes in V_r_ during a stimulus cycle, because blocking them increases the V_r_ values underlying the spiking activity [(25); Figures 6A,C,E]. Due to the dependency of E_Vr_ on V_r_ (Equation 1), these different inhibitory inputs reduce the amount of extra ATP required for the maintenance of V_r_ during the stimulation cycle (Figures 4A,C,E). This applies for local strychnine-sensitive glycinergic (Figures 4A,B,G) and bicuculline-sensitive GABAergic inputs (Figures 4C,D,G). In addition, inhibitory inputs from cerebellar Purkinje, which were blocked by injection of lidocaine into the cerebellum, exhibited the same changes (Figures 4E–G). The reduction in ATP consumption by local or cerebellar inhibitory shunting mechanisms (Figure 4G) was expressed as fraction of the non-inhibited value (Figure 4H). This allowed for a direct comparison of ATP saving by the different mechanisms. We did not take into account dynamic and temporal properties of inhibition. This simplification was based on the idea to keep the number of parameters for the calculations small (V_r_, R_in_, ΔAP and O). (2) Inhibitory (glycinergic and GABAergic) currents increase the membrane conductance (mainly for Cl^−^) and, as a consequence, R_in_ decreases and E_Vr_ increases. ATP saving by inhibition [see (1), Figure 4H] might counter-balance higher E_Vr_ values due to the change in R_in_, which becomes smaller in the presence of inhibitory currents. Based on Equation 1, a reduction of R_in_ by 10, 20% or 50% would result in an E_Vr_ increase by 11, 25 and 100%, respectively.

(3) The inhibitory inputs to phasic neurons essentially originate from local tonic type vestibular interneurons (19). Therefore, when calculating ATP consumption of phasic neurons during a temporally extended activation by vestibular nerve afferents, the energy consumption of tonic vestibular neurons must be considered in addition as a contributor to the neuronal computation of phasic vestibular neurons. Thus, based on the principal energy needs of the two subtypes (Figures 2A,B), the total ATP consumption of phasic neurons is even higher when the inhibition through local interneurons is included as cost factor to produce the highly dynamic AP pattern in phasic vestibular neurons. Dependent on which components are considered, this increase can be very different. Estimating the total ATP need of tonic neurons, an increase by about 50–60% appears to be realistic. However, when arguing that tonic vestibular neurons also serve as projection neurons, as complement to the phasic pathway, E_Vr_ can be omitted. In this case, only E_AP_ has to be considered which would increase the total ATP need by less than 5%. However, it is more likely that the inhibitory side loop within the vestibular nuclei is formed by local interneurons rather than by projection neurons with axon collaterals onto phasic neurons (19) arguing that the energetic cost for the inhibitory side loop forms in fact a considerable factor that must be added to the costs for neuronal processing in phasic vestibular neurons. This therefore suggests that the encoding of high dynamic head motion profiles is particularly cost-intensive at variance with the computation of slow or tonic head deviations.

## Discussion

The morpho-physiologically well studied two types of frog central vestibular neurons, responsible for encoding the large dynamic range of head/body motion, were used to estimate the energy consumption for AP generation and V_r_ maintenance during simulated natural inputs based on available physiological parameters. This approach benefitted from the distinct differences in action potential shape and firing patterns that caused in the two cell types a phasic and tonic spike discharge, respectively. A clear energetic difference was obtained by applying a calculation method that only required few biophysical and morphological parameters to calculate ATP consumption for the energetically most relevant neuronal processes: maintenance of resting membrane potential (V_r_) and generation of action potentials. Accordingly, the analysis demonstrated that signal processing of phasic vestibular neurons is considerably (two times) more cost-intensive compared to that of tonic vestibular neurons (Figure 2A), despite the production of substantially more action potentials in the latter type. This finding is similar to our calculation in auditory brainstem neurons (22), which share with vestibular neurons a low input resistance as major determinant for a high E_Vr_. The high ion permeability of their leaky plasma membranes (due to a high density of ion channels) would result in a constant and fast break-down of the ion gradients necessary for maintaining V_r_ if the Na^+^,K^+^-ATPAse would not work with a high rate. This is in contrast to ATP calculations in cortical neurons of the cerebrum or cerebellum with a much (one to two orders of magnitude) higher input resistance, which yielded a much larger fractional contribution of E_AP_.

### Methodological validity of calculating ATP consumption

The algorithms for calculating ATP consumption of various neuronal processes are well established and have been successfully applied to different neural systems (22–24, 28). Moreover, the electrophysiological and morphological parameters utilized for the calculations of ATP consumption were well known prior to our calculations [e.g., (5, 19–21)]. In addition, all calculations were performed on 1,000 individual neurons based on the mean values and variability of the employed parameters including a random combination of individual parameters (Figure 2). These data show entailed similar mean values as calculations based on mean parameters. However, this procedure also revealed a large variability between individual neurons. Thus, it would be interesting to evaluate whether this variability is a consequence of the random permutation of the parameters or an inherent variability. Experimentally, this would be rather laborious, because all necessary electrophysiological and morphological parameters would have to be obtained for each individual neuron.

As in previous studies (22–24), E_Vr_ and E_AP_ represented the two major energy-consuming processes in our calculations. However, a third process that turned out to substantially contribute to the overall energy consumption - but was distinctly neuronal type-dependent - was the amount of ATP required to restore trans-membranous Na^+^ gradients that have been perturbed by glutamatergic currents, which in the case of both types of central vestibular neurons were activated by the dominating monosynaptic afferent inputs from the inner ear sensory periphery (31). This fraction, which generally accounts for ~10% of the total ATP consumption in most neurons (22, 24, 28) was omitted because of the lack of specific electrophysiological parameters necessary for the calculation, i.e., decay constant of excitatory postsynaptic currents and threshold for AP generation. Assuming similar AP generation thresholds for the two vestibular neuronal types and the activation of EPSPs by afferent inputs at the level of the soma and proximal dendrites in both subtypes (31), differences in energy expedition due to different decay time constants of the EPSPs are possible but likely have an inferior impact with respect to those produced by E_Vr_ and E_AP_, demonstrated in the present study.

Our calculations represent an estimate of energy expenditure based on a simple approach, but relying on only a few experimentally well-known parameters. This simplification, however, neglects dynamic aspects regarding inhibition and synaptic plasticity, which would be interesting to analyze in future studies. Experimental evidence by monitoring changes in ATP and O_2_ concentrations could substantiate our calculations.

### Physiological relevance of energetic differences in the dual frequency-tuned pathways

The direct comparison of the two types of vestibular neurons on a single neuron basis clearly revealed that tonic neurons use less ATP than phasic vestibular neurons across their respective range of physiological activity (Figure 2). This result might seem at first glance counterintuitive, because a tonic spike firing profile should be more costly simply based on the exceedingly larger number of generated APs. However, neurons with a low or very low input resistance, such as phasic vestibular neurons (5, 20) use a major fraction of the available ATP to maintain the resting membrane potential. Accordingly, the total amount of ATP consumption of these neurons is less dominated by the frequency of AP generation, comparable to the situation found in auditory neurons, as described earlier (22). This indicates that even though phasic vestibular neurons produce considerably fewer spikes during, e.g., synaptic activation compared to tonic neurons, this sparseness in AP generation does not offset the extensive amount of ATP that is required to maintain the resting membrane potential in the phasic cell type. Even though the 4:1 ratio of frog phasic relative to tonic vestibular neurons (5) might be an overestimation due to a sampling bias for the recording of larger neurons, the population of phasic neurons likely constitutes the major cell type in this nucleus. This indicates that the energy expense for vestibular signal processing is dominated by phasic vestibular neurons. Assessing the total ATP consumption of the two neuron populations, however requires the knowledge of the approximate numbers of each neuronal type within the vestibular nucleus.

Of greater importance for the ATP consumption of the entire nucleus is the presence of cross-links between the two neuronal types through feedforward and feedback loops. Inhibitory tonic neurons shape the firing pattern of the phasic type (19). This modulatory influence is metabolically highly relevant and the ATP consumption by these inhibitory processes has to be considered in addition when calculating the amount of ATP expended in the circuit when involving phasic neurons. As inhibition in phasic neurons is provided by tonic neurons (19), an additional amount of ATP due to the activity of tonic neurons has to be taken into account. However, since maintaining E_Vr_ in tonic neurons is necessary for other features, i.e., tonic firing, only the additional ATP amount for AP generation is required, which however is relatively small (Figures 2A,G), nonetheless has to be added for the calculation of the costs of phasic neurons to process synaptic inputs.

On the other hand, inhibition as prerequisite for the phasic firing pattern in the respective neuronal type, might also be interpreted as means for saving energy. Our calculations have shown that the inhibition decreases a rise in V_r_ during each cycle (21) and thereby reduces E_Vr_ (Figure 4). Thus, the GABAergic inhibition, based on its voltage profile shows the strongest energy saving impact. However, the dependency of the energetic impact on the blocker concentration in pharmacological experiments would have required a more detailed knowledge of the outcome to compare the ATP savings quantitatively. These findings are also of general interest, since inhibitory neurons and related processes are so far assumed to be of only limited energetic relevance in many parts of the brain (23, 24). However, inhibition has a second consequence, which is often underestimated and seldom finds entry into calculations of the energetic profile of neuronal computations. Activated inhibitory currents cause a reduction of R_in_ to a yet unknown fraction. Hence, E_Vr_ will be raised (see Equation 1). Quantitative data on the R_in_ change - from experiments or modelling - will be necessary to assess the overall effect on E_Vr_ by the two processes, i.e., the change in V_r_ and in R_in_. As phasic neurons represent the dominant type in the vestibular nucleus (17), the energetic consequences of inhibition might be substantial. In summary, inhibition has rather complex metabolic consequences and needs further consideration when calculating its impact on energy metabolism.

### Evolutionary considerations

In pre-vertebrate ancestors, the peripheral vestibular system in the inner ear consisted of a simple gravistatic organ that detected low frequency passive head/body motion within the aquatic environment (1). Central processing of this motion dynamics required neurons that were able to linearly transform motion-related sensory signals into motor commands for slow postural and possibly also ocular adjustments, computational tasks for which tonic vestibular neurons are ideally suited. This suggests that this neuronal type represented the initial cellular substrate responsible for vestibular signal processing. Only later during vertebrate evolution, with the concurrent increase of locomotor capacity and maneuverability, semicircular canals appeared (32, 33). These latter sensory elements are particularly sensitive for high frequency head/body movements. In addition, central vestibular circuits with phasic vestibular neurons are required to enable the processing of such signals (2). While the correspondence between increased locomotor capacity and vestibular sensory organ radiation and specification has so far been assumed as the direct correlate for the necessity of a phasic central vestibular system, it might well be that the larger energetic costs of such a system has influenced the progression of its implementation. Thus, energy expedition, even though difficult to validate in hindsight, might represent a so far underestimated constraint that during evolution might have influenced the capacity of vertebrates to encode and process high dynamic head/body motion signals. This hypothesis is valid, if ATP expenditure for V_r_ maintenance is the dominating process as yielded by our calculations.

While all vertebrate species studied so far possess a set of central vestibular neurons, which are able to transform high dynamic signals (12), phasic vestibular neurons in adult frogs are unique in their excessive degree of spike rate adaptation (34). The presence of such highly phasic central vestibular neurons in adult frogs has been considered an eco-physiological adaptation to the movement strategy of these animals (12). Prey capture in frogs consists of brief, rapid orienting movements, interrupted by long periods of immobility (35). While for this “wait and catch” behavior a self-motion detection system with highly phasic vestibular neurons serving as event detectors is particularly suitable (12), such a system does not necessarily represent an energy-saving adaptation as demonstrated in the current study. The neuronal generation of event detectors for head/body motion in adult frogs, even though the implementation can afford a lower temporal precision than required for those in the auditory system (36), is energetically still rather cost-intensive. A similarly high energy cost is likely also to be expected for the even more leaky Mauthner cell (37). These singular neurons on each side of the hindbrain constitute typical event detectors for lateralized sensory inputs, initiating a startle response (C-start) with only very few APs transmitted to motoneurons in the contralateral spinal cord.

Extremely phasic neurons, as observed in adult frogs are absent from the vestibular nuclei of mammalian species such as guinea pigs or rats (12) even though similarly phasic neurons have been reported in the anatomically adjacent nucleus prepositus hypoglossi in the hindbrain of guinea pigs (38). However, the general lack of such neurons in the vestibular nuclei of mammals complies with the respective locomotor style that in these species essentially consists of constant, smooth movements. They may be best processed centrally by vestibular neurons with a continuous ongoing discharge that can be modulated over a large dynamic range. It is therefore possible that the encoding of such head/body motion signals is energetically cheaper than the central processing of the motion pattern of adult frogs, which consists of long immobile periods interrupted by sudden, fast movements. So, in contrast to the simple assumption that processing of immobility, as in lurking frogs, is cheap in energetic cost, it is rather not, because the provision of an event detector in case of a rapid movement is expensive as demonstrated for the population of phasic central vestibular neurons.

## Conclusion

The existence of two types of vestibular neurons in the frog renders this dual computational substrate an energetically interesting system. We suggest that the interpretation of functional neuro-imaging might also benefit from the knowledge of the metabolic diversity in different neuron types (39–41). Phasic vestibular neurons dominate the ATP expense for AP generation and V_r_ maintenance in circuits dealing with head/body movements. In addition, inhibition, which is often neglected with regard to its energetic contribution, plays an essential physiologic and metabolic part in the vestibular nucleus. Physiologically, since it contributes to shaping the phasic activity pattern, metabolically, because ATP consumption by inhibitory neurons adds to the total consumption of the nucleus, but also because of the potential energy saving impact as shown in our study. Assuming metabolic demand is dominated by maintaining V_r_ due to the low membrane resistance in both phasic and tonic vestibular neurons, ATP consumption might have been an evolutionary constraint for the ability to process dynamic motion signals.

## Data Availability

The data analyzed in this study is subject to the following licenses/restrictions: Data used for ATP calculations were either taken directly as mean +/− errors or obtained by digitization of graphs from original publications by the late Hans Straka. Requests to access these datasets should be directed to Lars Kunz, lars.kunz@bio.lmu.de.

## Footnotes

^1^https://www.digitizeit.xyz

## References

[1] Straka H Gordy C. The vestibular system: the “Leatherman™” among sensory systems In: Fritzsch B Straka H, editors. The senses: A comprehensive reference, vol. 6. Cambridge, MA: Elsevier, Academic Press (2020). 708–20.

[2] Fritzsch B Straka H. Evolution of mechanosensory hair cells and inner ears: identifying stimuli to select altered molecular development toward new morphologies. J Comp Physiol A. (2014) 200:5–18. doi: 10.1007/s00359-013-0865-z, PMID: 24281353 PMC3918741

[3] Goldberg JM. Afferent diversity and the organization of central vestibular pathways. Exp Brain Res. (2000) 130:277–97. doi: 10.1007/s002210050033, PMID: 10706428 PMC3731078

[4] Paulin MG Hoffman LF. Models of vestibular semicircular canal afferent neuron firing activity. J Neurophysiol. (2019) 122:2548–67. doi: 10.1152/jn.00087.2019, PMID: 31693427 PMC6966309

[5] Straka H Dieringer N. Basic organization principles of the VOR: lessons from frogs. Prog Neurobiol. (2004) 73:259–309. doi: 10.1016/j.pneurobio.2004.05.003 15261395

[6] Horn AKE. Neuroanatomy of central vestibular connections In: Fritzsch B Straka H, editors. The senses: A comprehensive reference, vol. 6. Cambridge, MA: Elsevier, Academic Press (2020). 21–37.

[7] Hänzi S Straka H. Developmental changes in head movement kinematics during swimming in *Xenopus laevis* tadpoles. J Exp Biol. (2017) 220:227–36. doi: 10.1242/jeb.146449, PMID: 27811303

[8] Carriot J Jamali M Chacron MJ Cullen KE. The statistics of the vestibular input experienced during natural self-motion differ between rodents and primates. J Physiol. (2017) 595:2751–66. doi: 10.1113/JP273734, PMID: 28083981 PMC5390882

[9] Carriot J Jamali M Chacron MJ Cullen KE. Statistics of the vestibular input experienced during natural self-motion: implications for neural processing. J Neurosci. (2014) 34:8347–57. doi: 10.1523/JNEUROSCI.0692-14.2014, PMID: 24920638 PMC4051983

[10] Sadeghi SG Chacron MJ Taylor MC Cullen KE. Neural variability, detection thresholds, and information transmission in the vestibular system. J Neurosci. (2007) 27:771–81. doi: 10.1523/JNEUROSCI.4690-06.2007, PMID: 17251416 PMC5053814

[11] Straka H Lambert FM Pfanzelt S Beraneck M. Vestibulo-ocular signal transformation in frequency-tuned channels. Ann N Y Acad Sci. (2009) 1164:37–44. doi: 10.1111/j.1749-6632.2008.03740.x 19645878

[12] Straka H Vibert N Vidal PP Moore LE Dutia MB. Intrinsic membrane properties of vertebrate vestibular neurons: function, development and plasticity. Prog Neurobiol. (2005) 76:349–92. doi: 10.1016/j.pneurobio.2005.10.002, PMID: 16263204

[13] Serafin M de Waele C Khateb A Vidal PP Mühlethaler M. Medial vestibular nucleus in the guinea-pig. I. Intrinsic membrane properties in brainstem slices. Exp Brain Res. (1991) 84:417–25. doi: 10.1007/BF00231464 2065749

[14] Johnston AR MacLeod NK Dutia MB. Ionic conductances contributing to spike repolarization and afterpotentials in rat medial vestibular nucleus neurones. J Physiol. (1994) 481:61–77. doi: 10.1113/jphysiol.1994.sp020419, PMID: 7531769 PMC1155866

[15] Shimazu H Precht W. Tonic and kinetic responses of cat’s vestibular neurons to horizontal angular acceleration. J Neurophysiol. (1965) 28:991–1013. doi: 10.1152/jn.1965.28.6.991, PMID: 5295930

[16] Peusner KD Giaume C. Ontogeny of electrophysiological properties and dendritic pattern in second-order chick vestibular neurons. J Comp Neurol. (1997) 384:621–33. doi: 10.1002/(SICI)1096-9861(19970811)384:4<621::AID-CNE9>3.0.CO;2-4, PMID: 9259493

[17] Straka H Beraneck M Rohregger M Moore LE Vidal PP Vibert N. Second-order vestibular neurons form separate populations with different membrane and discharge properties. J Neurophysiol. (2004) 92:845–61. doi: 10.1152/jn.00107.2004 15044516

[18] Straka H Holler S Goto F. Patterns of canal and otolith afferent input convergence in frog second order vestibular neurons. J Neurophysiol. (2002) 88:2287–301. doi: 10.1152/jn.00370.2002, PMID: 12424270

[19] Rössert C Moore LE Straka H Glasauer S. Cellular and network contributions to vestibular signal processing: impact of ion conductances, synaptic inhibition and noise. J Neurosci. (2011) 31:8359–72. doi: 10.1523/JNEUROSCI.6161-10.2011, PMID: 21653841 PMC6623320

[20] Beraneck M Pfanzelt S Vassias I Rohregger M Vibert N Vidal PP. Differential intrinsic response dynamics determine synaptic signal processing in frog vestibular neurons. J Neurosci. (2007) 27:4283–96. doi: 10.1523/JNEUROSCI.5232-06.2007, PMID: 17442812 PMC6672329

[21] Pfanzelt S Rössert C Rohregger M Glasauer S Moore LE Straka H. Differential dynamic processing of afferent signals in frog tonic and phasic second-order vestibular neurons. J Neurosci. (2008) 28:10349–62. doi: 10.1523/JNEUROSCI.3368-08.2008, PMID: 18842894 PMC6671017

[22] Trattner B Gravot CM Grothe B Kunz L. Metabolic maturation of auditory neurones in the superior olivary complex. PLoS One. (2013) 8:e67351. doi: 10.1371/journal.pone.0067351, PMID: 23826275 PMC3694961

[23] Attwell D Laughlin SB. An energy budget for signaling in the grey matter of the brain. J Cereb Blood Flow Metab. (2001) 21:1133–45. doi: 10.1097/00004647-200110000-00001, PMID: 11598490

[24] Howarth C Gleeson P Attwell D. Updated energy budgets for neural computation in the neocortex and cerebellum. J Cereb Blood Flow Metab. (2012) 32:1222–32. doi: 10.1038/jcbfm.2012.35, PMID: 22434069 PMC3390818

[25] Brosel S Grothe B Kunz L. An auditory brainstem nucleus as a model system for neuronal metabolic demands. Eur J Neurosci. (2018) 47:222–35. doi: 10.1111/ejn.13789 29205598

[26] Gleiss H Encke J Lingner A Jennings TR Brosel S Kunz L. Cooperative population coding facilitates efficient sound-source separability by adaptation to input statistics. PLoS Biol. (2019) 17:e3000150. doi: 10.1371/journal.pbio.3000150, PMID: 31356637 PMC6687189

[27] Grafe P Rimpel J Reddy MM ten Bruggencate G. Changes of intracellular sodium and potassium ion concentrations in frog spinal motoneurons induced by repetitive synaptic stimulation. Neuroscience. (1982) 7:3213–20. doi: 10.1016/0306-4522(82)90243-3, PMID: 6984493

[28] Nawroth JC Greer CA Chen WR Laughlin SB Shepherd GM. An energy budget for the olfactory glomerulus. J Neurosci. (2007) 27:9790–800. doi: 10.1523/JNEUROSCI.1415-07.2007, PMID: 17804639 PMC6672954

[29] Straka H Dieringer N. Convergence pattern of uncrossed excitatory and inhibitory semicircular canal-specific inputs onto second-order vestibular neurons of frogs. Exp Brain Res. (2000) 135:462–73. doi: 10.1007/s002210000544 PMID: 11156310

[30] Biesdorf S Malinvaud D Reichenberger I Pfanzelt S Straka H. Differential inhibitory control of semicircular canal nerve afferent-evoked inputs in second-order vestibular neurons by glycinergic and GABAergic circuits. J Neurophysiol. (2008) 99:1758–69. doi: 10.1152/jn.01207.2007, PMID: 18256163

[31] Straka H Biesdorf S Dieringer N. Canal-specific excitation and inhibition of frog second-order vestibular neurons. J Neurophysiol. (1997) 78:1363–72. doi: 10.1152/jn.1997.78.3.1363 9310427

[32] Retzius A. Das Gehörorgan der Wirbelthiere: morphologisch-histologische Studien (I.): Das Gehörorgan der Fische und Amphibien. Stockholm: Samson and Wallin (1881).

[33] Fritzsch B. Evolution of the vestibulo-ocular system. Otolaryngol Head Neck Surg. (1998) 119:182–92. doi: 10.1016/S0194-5998(98)70053-1 9743074

[34] Beraneck M Straka H. Vestibular signal processing by separate sets of neuronal filters. J Vest Res. (2011) 21:5–19. doi: 10.3233/VES-2011-0396, PMID: 21422539

[35] Schneider D. Das Gesichtsfeld und der Fixiervorgang bei einheimischen Anuren. Z Vergl Physiol. (1954) 36:147–64. doi: 10.1007/BF00297744

[36] Grothe B Pecka M McAlpine D. Mechanisms of sound localization in mammals. Physiol Rev. (2010) 90:983–1012. doi: 10.1152/physrev.00026.2009 20664077

[37] Nakayama H Oda Y. Common sensory inputs and differential excitability of segmentally homologous reticulospinal neurons in the hindbrain. J Neurosci. (2004) 24:3199–209. doi: 10.1523/JNEUROSCI.4419-03.2004, PMID: 15056699 PMC6730040

[38] Idoux E Serafin M Fort P Vidal PP Beraneck M Vibert N. Oscillatory and intrinsic membrane properties of guinea pig nucleus prepositus hypoglossi neurons in vitro. J Neurophysiol. (2006) 96:175–96. doi: 10.1152/jn.01355.2005, PMID: 16598060

[39] Shulman RG Hyder F Rothman DL. Insights from Neuroenergetics into the interpretation of functional neuroimaging: an alternative empirical model for studying the Brain’s support of behavior. J Cereb Blood Flow Metab. (2014) 34:1721–35. doi: 10.1038/jcbfm.2014.145, PMID: 25160670 PMC4269754

[40] Thompson GJ Riedl V Grimmer T Drzezga A Herman P Hyder F. The whole-brain "global" signal from resting state fMRI as a potential biomarker of quantitative state changes in glucose metabolism. Brain Connect. (2016) 6:435–47. doi: 10.1089/brain.2015.0394, PMID: 27029438 PMC4976226

[41] Mortensen KN Gjedde A Thompson GJ Herman P Parent MJ Rothman DL. Impact of global mean normalization on regional glucose metabolism in the human brain. Neural Plast. (2018) 2018:1–16. doi: 10.1155/2018/6120925 PMC6020504 30008742

